# Urinary Sweeteners and Sugars in Relation to Childhood Obesity: The SWEET Project

**DOI:** 10.1016/j.tjnut.2025.10.019

**Published:** 2025-10-10

**Authors:** Xinyi Cai, Elske Brouwer-Brolsma, Gerben de Gier, Novita D Naomi, Joanne A Harrold, Jason CG Halford, Anne Raben, Michiel Balvers, Edith JM Feskens

**Affiliations:** 1 Division of Human Nutrition and Health, Wageningen University & Research, Wageningen, The Netherlands; 2 Department of Psychology, Institute of Population Health, University of Liverpool, Liverpool, United Kingdom; 3 School of Psychology, Faculty of Medicine & Health, University of Leeds, Leeds, United Kingdom; 4 Department of Nutrition, Exercise and Sports, University of Copenhagen, Copenhagen, Denmark

**Keywords:** childhood obesity, biomarkers, sweeteners, sugars, metabolic health

## Abstract

**Background:**

The roles of low-and no-calorie sweeteners (LNCS) and sugars in obesity remain debated, partly because of the limitations of self-reported dietary intakes.

**Objectives:**

To examine cross-sectional associations between urinary LNCS and sugar concentrations and BMI and waist-to-height (WHtR) z-scores in Dutch children and adolescents.

**Methods:**

We used data and urine samples of 500 participants aged 8–17 y sampled from the Lifelines Cohort. LNCS and sugar concentrations were measured from timed overnight urine samples using ultra-pressure liquid chromatography coupled to tandem mass spectrometry. Multivariable linear regression models assessed associations of urinary LNCS and sugars (mg/mL or log-transformed urinary biomarker-to-creatinine ratio) with BMI and WHtR z-scores, adjusting for age, sex, screen time hours, and fruit and vegetable frequencies of use.

**Results:**

The median age of the study population was 14 y, and 50% (*n =* 249) were girls. Mean BMI z-score amounted to 0.10 (SD: 1.0) and WHtR z-score was 0.16 (SD: 1.0). Urinary saccharin was independently associated with BMI z-score {β_mg/mL_: 51.59 [95% confidence interval (CI): 7.96, 95.21]}, and urinary sucralose with both BMI z-score [β_mg/mL_: 291.29 (95% CI: 98.01, 484.58)] and WHtR z-score [β_mg/mL_: 238.75 (95% CI: 44.53, 432.98)]. Inverse associations with BMI z-score were observed for urinary sugar concentrations: total sugar, fructose, glucose, and similarly for WHtR z-score. BMI and WHtR z-scores were not significantly associated with urinary sucrose, total urinary LNCS, acesulfame, cyclamate, and steviol.

**Conclusions:**

Urinary saccharin and sucralose were positively associated with indicators of general and abdominal obesity in children and adolescents, whereas total urinary sugar, glucose, and fructose showed inverse associations. These findings likely reflect differences in dietary patterns of children and adolescents according to weight status, as misreporting can be excluded because of the use of biomarkers.

## Introduction

Childhood obesity has become a major public health challenge [1]. In 2022, ∼18.9% of children and adolescents aged 5–19 y worldwide were affected by overweight or obesity, including 7.2% with obesity [2]. As childhood obesity often persists into adulthood, thereby increasing risk of chronic diseases and mortality [3], identifying modifiable factors during childhood, such as diet, is crucial to address the growing prevalence of overweight and obesity.

Sugars and sweeteners have received increasing attention in this respect. In the Netherlands, foods contributing to sugar intake among children (aged 7–18 y) are honey/jams, confectionery, chocolate, cake, cookies, dairy, and sugar-sweetened beverages (SSBs) [4]. In particular, sugars from SSBs have been consistently linked to obesity risk in children and adolescents because of their low satiety and incomplete energy compensation [5,6]. A systematic review and meta-analysis including 40 cohort studies showed that each additional serving of SSB (355 mL) was associated with a 0.07 kg/m^2^ increase in BMI among children and adolescents aged below 18 y [7]. The WHO, therefore, suggested reducing sugar intake to <10% of daily energy intake [8]. In response, food products containing low-and no-calorie sweeteners (LNCS) have become increasingly popular as a replacement [9]. These LNCS can be classified as either artificial or natural sweeteners and exclude sugar alcohols (polyols) such as xylitol, which provide measurable caloric content (2.4 kcal/g). Although it has been suggested that LNCS could be used for weight management [10], the direction of their association with obesity remains controversial. A meta-analysis of 13 cohort studies showed that LNCS consumption was associated with higher BMI among children and adolescents ranging from 2 to 17 y old {odds ratio 1.15 [95% confidence interval (CI): 1.06, 1.25]} [11]. This may be attributed to reverse causality, that is, individuals with overweight or obesity may prefer LNCS over sugars, resulting in observed positive associations [12].

Other observational studies in children and adolescents have also explored low-and no-calorie sweetened beverages (LNCSBs) and SSBs, and their associations with obesity indicators, including BMI [[13], [14], [15], [16], [17], [18], [19], [20], [21], [22], [23]] and waist-to-height ratio (WHtR) [[18], [19], [20]], a proxy of central fat distribution [24]. In these studies, intakes of LNCS and sugars were indirectly estimated through self-reported beverages consumption using 24-h dietary recalls [13,14], brief questionnaires [15], food frequency questionnaires [[16], [17], [18], [19], [20]], and food records [[21], [22], [23]]. Although large-scale studies benefit from the cost-effectiveness of these dietary assessment tools, often they are subjective to errors such as recall bias, unawareness of hidden ingredients, socially desirable reporting, and incomplete food composition databases [25].

In contrast, nutritional biomarkers provide more objective measures of dietary exposure [25]. Recently, new analytical methods have enabled the quantification of major LNCS and sugars in blood and urine [[26], [27], [28]]. Further work has shown that urinary excretion of LNCS and sugars has the potential to serve as measures of intake, with improved performance over self-reported assessments [29]. In this context, the application of biomarkers is expected to provide novel insights into the associations of LNCS and sugars with obesity risk by directly assessing their overall dietary exposure, beyond beverage sources and avoiding misreporting. Therefore, this study aimed to investigate the associations of LNCS and sugar excretion with standardized BMI and WHtR among Dutch children and adolescents, using nutritional biomarkers as objective measures of dietary exposure and taking into account whether these associations differ by age and sex subgroups [[30], [31], [32], [33]].

## Methods

### Study design

Lifelines is a multidisciplinary population-based cohort study examining in a unique 3-generation design the health and health-related behaviors of 167,729 persons aged between 6 mo and 93 y living in the North of the Netherlands [34]. It employs a broad range of investigative procedures in assessing the biomedical, sociodemographic, behavioral, physical, and psychological factors that contribute to the health and disease of the general population, with a special focus on multimorbidity and complex genetics. Participants were registered through their general practitioners, or they could self-register via the Lifelines website. Children and adolescents in this cohort were recruited when one of their parents was a participant. Of the 167,729 participants who agreed to participate in Lifelines, 14,801 were under the age of 18 y. Lifelines was approved by the Medical Ethical Committee of the University Medical Center Groningen (UMCG), the Netherlands (2007/152); and was conducted according to the principles of the Declaration of Helsinki and the research code UMCG [34].

On the basis of feasibility and resources, 500 participants were selected from the Lifelines Cohort for urinary assessment, with random sampling of *n* = 250 carried out in 2 age strata, that is, ages 8–14 and 15–17 y, using SPSS. During the third assessment of Lifelines (“3A,” between 2019 and 2023), participants who provided informed consent were invited to 1 of the 12 research sites, where physical examination was conducted for anthropometric measurements. Before the first visit, parents or adolescents (aged 13–17 y) received Lifelines questionnaires by mail specific to age. After 2 wk, a second visit was arranged for the collection of a single timed overnight urine sample from children and adolescents. Upon arrival at the Lifelines laboratory, samples were weighed and transferred into BD evacuated tube urine tubes (without additives or preservatives). Urine aliquots were first refrigerated at 4°C and subsequently stored at −80°C (within 10 h after sample collection) until further analysis [35].

### Determination of urinary LNCS and sugar concentrations

Exposures include concentrations of urinary LNCS and sugars, that is, acesulfame, cyclamate, sucralose, saccharin, steviol glucuronide, glucose, fructose, and sucrose, which were determined in our laboratory using a modified protocol of a previously published ultra-pressure liquid chromatography coupled to tandem mass spectrometry (UPLC-MS/MS) method [27]. The modifications involved the use of a new UPLC column for chromatography, whereas the rest of the method remained unchanged from the originally published protocol. Urine samples were thawed at 5°C and diluted 20-fold with a mixture containing internal standards, ammonia, methanol, and ULC/MS grade water. Chromatographic separation was achieved on a Shodex Asahipak NH2P-50 2D column (Showa Denko Europe GmbH, Munich, Germany; 150 × 2.0 mm, 4 μm), which was kept at 55 C. The mobile phases consisted of (A) 1.0 mM ammonium acetate and 0.1% (vol:vol) ammonia in ULC/MS grade water, and (B) 100% acetonitrile, with a flow rate of 0.25 mL/min and gradient elution. The UPLC-MS/MS system consisted of an Acquity H-class Plus UPLC coupled to a Xevo TQ-S micro tandem mass spectrometer (Waters Chromatography Europe BV) with electrospray ionization in negative ion mode and selective reaction monitoring. The limits of detection and limits of quantification for the measured LNCS and sugars have been previously described [27].

Total LNCS and sugars concentrations were calculated as the sum of their respective components. To address potential variation in urine dilution between individuals, urinary biomarker-to-creatinine ratio (UBCR) was obtained by dividing urinary biomarker concentration by urinary creatinine concentration [36]. Urinary creatinine concentration was measured using a Roche Enzymatic assay at the UMCG laboratory [35].

### Obesity outcomes

Height (without shoes) was measured on site using a portable stadiometer (SECA 222), weight (without shoes and heavy clothing) with an electronic weighing scale (SECA 761), and waist circumference with a flexible tape (SECA 200), all by trained research nurses. BMI was calculated from height and weight to assess general obesity, whereas WHtR was derived as waist circumference (cm) divided by height (cm) to indicate central obesity [24]. To enable standardized comparison of different obesity parameters across children and adolescents of different ages and sexes, BMI and WHtR were both converted into age- and sex-specific z-scores as outcomes of the study [[37], [38], [39], [40]]. For descriptive purposes, BMI was further categorized as overweight (1 ≤ BMI z-score < 2) and obesity (BMI z-score ≥ 2) [40]; and central obesity was defined as WHtR ≥0.5 [24].

### Other variables

Age (y), sex (boys, girls), screen time (h/d), and growth spurt stages (not started, just started, started some time ago, finished) were assessed using Lifelines questionnaires [35,41].

Frequencies of fruit, vegetable, soft drink, and sweet snack consumption were self-reported using a food frequency questionnaire [35,42]. Response categories included “never,” “less than once a week,” “once a week,” “2 to 4 days a week,” “5 to 6 days a week,” “once every day,” and “more than once a day.” These frequencies were further dichotomized into “below 5 days per week” and “5 or more days per week” to reflect occasional compared with frequent consumption.

To calculate total soft drink intake, the categories “less than once a week” and “once a week” were coded as 1 d/wk, and ranges for other categories were coded to the midpoint of their respective ranges. These frequencies were multiplied by the mean number of glasses consumed per day to estimate total weekly servings, which were then divided by 7 to obtain daily intake. One serving was defined as one glass, can, or 250 mL.

### Statistical analysis

General characteristics of participants were stratified by the tertiles of total LNCS and total sugar concentrations (μg/mL) and expressed as mean with SD, median with IQR, or frequency with percentage. Total soft drink intake, sweet snack frequency, and growth spurt stage were described because of their relevance to this study, although not considered as confounders. Comparisons were performed using the ANOVA or Kruskal–Wallis test for continuous variables (depending on the normality of distribution), or chi-square test for categorical variables. BMI z-score and WHtR z-score were further stratified by age and sex groups.

For the primary analyses, linear regression models were used to assess the cross-sectional associations between urinary LNCS and sugars and obesity outcomes. Exposures included total LNCS, acesulfame, cyclamate, sucralose, saccharin, steviol glucuronide, as well as total sugars, glucose, fructose, and sucrose. Outcomes included BMI z-score and WHtR z-score; all of which were entered as continuous variables. To account for urine dilution and data skewness, urinary LNCS and sugar concentrations were modeled as separate terms in regression analyses, with UBCR and log-transformed UBCR in addition to the concentration in mg/mL. Crude and multivariable models, adjusted for age, sex, fruit and vegetable consumption frequencies, and screen time [[43], [44], [45], [46]], were fitted. All models were evaluated for linearity, independence, normality, and homoscedasticity.

To visualize potential nonlinear associations, adjusted restricted cubic splines (RCS) with 3 knots were fitted for associations of LNCS and sugars with BMI and WHtR z-scores. Scaled concentrations were used for better interpretability. The likelihood ratio test was applied to compare the linear model with the RCS model to determine *P* value for nonlinearity. We further conducted exploratory subgroup analyses to investigate whether the associations of total LNCS and sugars with BMI and WHtR z-scores differed by age and sex. *P* values for interaction were tested by introducing product terms in the regression models.

Because of the exploratory nature of our study, no corrections for multiple comparisons were made [47]. All statistical analyses were conducted in RStudio (version 2023.12.0 + 369) with R 4.4.0 (https://www.r-project.org/).

## Results

Table 1 presents the median and IQR of LNCS and sugar concentrations and their corresponding UBCR values. The median concentration of total LNCS was 10.7 μg/mL (IQR: 3.75–24.1), with a median UBCR of 8.28 (IQR: 2.82–17.4). For total sugar, the median concentration was 200 μg/mL (IQR: 120–322), and the median UBCR was 132 (IQR: 92.3–212).

**TABLE 1:** Urinary LNCS and sugar concentrations and UBCR values.

	No. of participants with 0 excretion^1^	Untransformed concentration^2^^,^^3^ (μg/mL)	UBCR^2^^,^^4^
Creatinine	—	1.42 [0.994–2.02]	—
Total LNCS^5^	—	10.7 [3.75–24.1]	8.28 [2.82–17.4]
Acesulfame	38	0.437 [0.00498–5.44]	0.321 [0.00359–4.14]
Saccharin	2	0.144 [0.0463–0.481]	0.106 [0.0309–0.365]
Sucralose	22	0.0636 [0.00774–0.248]	0.0480 [0.00669–0.181]
Cyclamate	58	0.00889 [0.00776–0.332]	0.00865 [0.00463–0.239]
Steviol glucuronide	0	4.81 [1.24–13.8]	3.72 [0.965–9.60]
Total sugar^6^	—	200 [120–322]	132 [92.3–212]
Glucose	0	75.8 [50.4–110]	51.5 [43.0–63.7]
Fructose	0	95.5 [49.8–187]	69.8 [34.8–137]
Sucrose	0	8.14 [4.01–18.1]	6.40 [3.17–11.4]

Abbreviations: LNCS, low-and no-calorie sweeteners; UBCR, urinary biomarker-to-creatinine ratio.

1 Zero excretion indicates undetectable levels but does not necessarily mean LNCS and sugars were absent in the samples.

2 Data are median [IQR].

3 Urinary concentrations are reported in μg/mL. For comparison with regression analyses, 1 μg/mL = 0.001 mg/mL.

4 UBCR is a unitless ratio, calculated by dividing urinary biomarker concentration (μg/mL) by urinary creatinine concentration (μg/mL).

5 Total LNCS = acesulfame + saccharin + sucralose + cyclamate + steviol glucuronide.

6 Total sugar = glucose + fructose + sucrose.

The study population had a median age of 14 y [IQR: 11–16], with 50% (*n* = 249) being girls (Table 2). Mean BMI z-score and WHtR z-score were 0.10 (SD: 1.0) and 0.16 (SD: 1.0), respectively. Specifically, 18.6% of participants were classified as overweight and 2.8% as obese (Supplemental Table 1), with a higher overweight rate (18.6% compared with 9.3%) but lower obesity rate (2.8% compared with 4.1%) than the Dutch national estimate for aged 4–20 y in 2023 [48]. Girls had a higher mean BMI z-score than boys [0.22 (SD: 0.9) compared with −0.004 (SD: 1.1), *P* value = 0.02]. In addition, 5.4% of the participants were classified as centrally obese.

**TABLE 2:** General characteristics of participants stratified by tertiles of total urinary LNCS and sugar concentrations.

	All^1^	Total urinary LNCS concentration^2^				Total sugar concentration^3^			
		Tertile 1	Tertile 2	Tertile 3	*P* value^4^	Tertile 1	Tertile 2	Tertile 3	*P* value^4^
No. of participants	500	165	165	170		165	165	170	
Demographic									
Age (y)	14 [11–16]	15 [11–16]	13 [11–16]	12 [11–15]	**0.01**	13 [11–16]	15 [11–16]	13 [11–16]	0.88
Sex (%)									
Girls	249 (49.8)	94 (57.0)	82 (49.7)	73 (42.9)	**0.04**	94 (57.0)	80 (48.5)	75 (44.1)	0.06
Boys	251 (50.2)	71 (43.0)	83 (50.3)	97 (57.1)		71 (43.0)	85 (51.5)	95 (55.9)	
Weight (kg)	54 (15.8)	55 (16.6)	53 (14.5)	53 (16.3)	0.66	54 (15.8)	54 (16.4)	53 (15.3)	0.77
Height (cm)	164 (15.1)	165 (15.1)	164 (14.5)	164 (15.7)	0.77	164 (14.9)	164 (15.1)	165 (15.3)	0.77
Waist circumference (cm)	68 (9.1)	68 (9.2)	68 (7.8)	68 (10.1)	0.83	69 (8.5)	68 (9.3)	67 (9.3)	0.24
Outcomes									
BMI z-score	0.10 (1.0)	0.01 (1.0)	0.14 (1.0)	0.16 (1.1)	0.38	0.19 (1.1)	0.13 (1.1)	−0.01 (0.9)	0.19
WHtR z-score	0.16 (1.0)	0.15 (0.9)	0.12 (0.9)	0.20 (1.2)	0.77	0.29 (1.0)	0.22 (1.0)	−0.03 (1.1)	**0.01**
Covariates									
Fruit frequency (%)									
<5 d/wk	150 (30.0)	41 (24.8)	48 (29.1)	61 (35.9)	0.08	43 (26.1)	55 (33.3)	52 (30.6)	0.35
≥5 d/wk	350 (70.0)	124 (75.2)	117 (70.9)	109 (64.1)		122 (73.9)	110 (66.7)	118 (69.4)	
Vegetable frequency (%)									
<5 d/wk	58 (11.6)	8 (4.8)	20 (12.1)	30 (17.6)	**0.001**	17 (10.3)	18 (10.9)	23 (13.5)	0.62
≥5 d/wk	442 (88.4)	157 (95.2)	145 (87.9)	140 (82.4)		148 (89.7)	147 (89.1)	147 (86.5)	
Screen time per day (h)	7.4 (3.3)	7.8 (3.4)	7.2 (3.4)	7.3 (3.1)	0.18	7.6 (3.4)	7.4 (3.2)	7.3 (3.2)	0.70
Other variables									
Total soft drinks intake^5^ (servings/d)	0.8 [0.4–2.0]	0.8 [0.1–1.6]	0.8 [0.4–2.0]	1.6 [0.8–3.0]	**<0.0001**	0.8 [0.3–1.6]	1.0 [0.4–2.0]	1.0 [0.4–2.3]	**0.02**
Sweet snack frequency (%)									
<5 d/wk	153 (30.6)	49 (29.7)	50 (30.3)	54 (31.8)	0.91	48 (29.1)	52 (31.5)	53 (31.2)	0.87
≥5 d/wk	347 (69.4)	116 (70.3)	115 (69.7)	116 (68.2)		117 (70.9)	113 (68.5)	117 (68.8)	
Growth spurt stage (%)									
Not started	110 (22.5)	36 (22.4)	41 (25.2)	33 (20.0)	**0.008**	36 (22.2)	40 (24.8)	34 (20.5)	0.09
Just started	63 (12.9)	16 (9.9)	24 (14.7)	23 (13.9)		30 (18.5)	15 (9.3)	18 (10.8)	
Started some time before	167 (34.2)	45 (28.0)	50 (30.7)	72 (43.6)		45 (27.8)	55 (34.2)	67 (40.4)	
Finished	149 (30.5)	64 (39.8)	48 (29.4)	37 (22.4)		51 (31.5)	51 (31.7)	47 (28.3)	

Abbreviations: IQR, interquartile range; LNCS, low-and no-calorie sweeteners; WHtR, waist-to-height ratio.

1 Data are mean (SD), median [IQR], or *n* (%).

2 Total LNCS = acesulfame + saccharin + sucralose + cyclamate + steviol glucuronide. Tertile 1, <5.83 μg/mL; tertile 2, 5.83–18.2 μg/mL; tertile 3, ≥18.2 μg/mL.

3 Total sugar = glucose + fructose + sucrose. Tertile 1, <143 μg/mL; tertile 2, 143–261 μg/mL; tertile 3, ≥261 μg/mL.

4 *P* value derived from ANOVA (continuous variables), Kruskal–Wallis Test (continuous variables, when displayed median values), or chi-square test (categorical variables).

5 One serving referred to 1 glass/can/250 mL/d.

The median total soft drink intake was 0.8 [IQR: 0.4–2.0] servings per day, and ∼70% of the participants consumed sweet snacks >5 d/wk (Table 2). Compared with those with lower total urinary LNCS concentrations, participants with higher LNCS concentrations were less likely to be girls, more likely to be younger, consumed vegetables less frequently, had higher total soft drink intake, and had a lower percentage who had completed their growth spurt. Participants in the highest tertile of total urinary sugar concentration were more likely to have a lower WHtR z-score and higher soft drink intake than those in the lowest tertile.

Crude and adjusted associations of urinary LNCS and sugars with BMI and WHtR z-scores are shown in Table 3. Regarding urinary LNCS, saccharin and sucralose were positively associated with BMI z-score before and after adjusting for confounders [saccharin: β_mg/mL_: 51.59 (95% CI: 7.96, 95.21); sucralose: β_mg/mL_: 291.29 (95% CI: 98.01, 484.58) and β_UBCR_: 0.30 (95% CI: 0.016, 0.59)]. For WHtR z-score, only sucralose was significantly associated after adjustments [β_mg/mL_: 238.75 (95% CI: 44.53, 432.98); β_UBCR_: 0.31 (95% CI: 0.019, 0.60)]. Total LNCS and individual urinary concentrations of acesulfame, cyclamate, and steviol were not significantly associated with either BMI or WHtR z-scores.

**TABLE 3:** Associations for urinary LNCS and sugar with BMI z-score and WHtR z-score.

	Model terms	BMI z-score		WHtR z-score	
		Crude model	Adjusted model^1^	Crude model	Adjusted model^1^
		β [95% CI]	β [95% CI]	β [95% CI]	β [95% CI]
Total LNCS^2^	Scaled (mg/mL)^3^	1.54 [−2.22, 5.31]	1.57 [−2.23, 5.37]	0.48 [−3.26, 4.22]	0.90 [−2.91, 4.71]
	UBCR^4^	0.0013 [−0.0042, 0.0068]	0.00060 [−0.0051, 0.0063]	0.0012 [−0.0043, 0.0067]	0.0023 [−0.0034, 0.0080]
	Log (UBCR + 1)^5^	0.059 [−0.029, 0.15]	0.052 [−0.040, 0.14]	0.025 [−0.063, 0.11]	0.047 [−0.045, 0.14]
Acesulfame	Scaled (mg/mL)	−4.00 [−11.04, 3.03]	−3.48 [−10.51, 3.56]	−2.32 [−9.31, 4.68]	−1.88 [−8.93, 5.18]
	UBCR	−0.0066 [−0.017, 0.0036]	−0.0069 [−0.017, 0.0033]	−0.0029 [−0.013, 0.0073]	−0.0018 [−0.012, 0.0084]
	Log (UBCR + 1)	0.0013 [−0.083, 0.086]	0.0033 [−0.082, 0.088]	0.023 [−0.061, 0.11]	0.032 [−0.054, 0.12]
Saccharin	Scaled (mg/mL)	**54.77 [11.13, 98.40]**	**51.59 [7.96, 95.21]**	32.43 [−11.07, 75.92]	34.95 [−8.89, 78.79]
	UBCR	**0.061 [0.0016, 0.12]**	0.055 [−0.0045, 0.11]	0.037 [−0.022, 0.096]	0.043 [−0.017, 0.10]
	Log (UBCR + 1)	**0.21 [0.0049, 0.41]**	0.19 [−0.016, 0.40]	0.10 [−0.10, 0.30]	0.13 [−0.078, 0.34]
Sucralose	Scaled (mg/mL)	**252.34 [60.27, 444.42]**	**291.29 [98.01, 484.58]**	**212.76 [21.72, 403.81]**	**238.75 [44.53, 432.98]**
	UBCR	0.28 [−0.0048, 0.56]	**0.30 [0.016, 0.59]**	0.26 [−0.024, 0.54]	**0.31 [0.019, 0.60]**
	Log (UBCR + 1)	0.42 [−0.054, 0.90]	0.46 [−0.028, 0.94]	0.25 [−0.22, 0.73]	0.34 [−0.15, 0.83]
Cyclamate	Scaled (mg/mL)	12.06 [−0.26, 24.37]	11.33 [−0.96, 23.62]	9.15 [−3.10, 21.40]	9.60 [−2.72, 21.93]
	UBCR	0.012 [−0.0069, 0.030]	0.0095 [−0.0089, 0.028]	0.0083 [−0.010, 0.026]	0.0094 [−0.0091, 0.028]
	Log (UBCR + 1)	0.068 [−0.054, 0.19]	0.066 [−0.056, 0.19]	0.063 [−0.058, 0.18]	0.070 [−0.052, 0.19]
Steviol glucuronide	Scaled (mg/mL)	2.68 [−3.28, 8.64]	2.54 [−3.49, 8.56]	−0.07 [−5.99, 5.85]	0.45 [−5.60, 6.49]
	UBCR	0.0044 [−0.0049, 0.014]	0.0034 [−0.0062, 0.013]	0.0025 [−0.0067, 0.012]	0.0042 [−0.0054, 0.014]
	Log (UBCR + 1)	0.038 [−0.051, 0.13]	0.024 [−0.068, 0.12]	0.0034 [−0.085, 0.092]	0.018 [−0.075, 0.11]
Total sugar^6^	Scaled (mg/mL)	−**0.33 [**−**0.57,** −**0.09]**	−**0.32 [**−**0.57,** −**0.08]**	−0.21 [−0.45, 0.035]	−0.20 [−0.44, 0.048]
	UBCR	−**0.00034 [**−**0.00062,** −**0.000061]**	−**0.00036 [**−**0.00064,** −**0.000082]**	−0.00019 [−0.00047, 0.000087]	−0.00016 [−0.00045, 0.00012]
	Log (UBCR + 1)	−**0.21 [**−**0.34,** −**0.076]**	−**0.24 [**−**0.37,** −**0.11]**	−**0.18 [**−**0.31,** −**0.055]**	−**0.18 [**−**0.31,** −**0.041]**
Glucose	Scaled (mg/mL)	−1.39 [−2.95, 0.17]	−1.31 [−2.88, 0.25]	−1.53 [−3.07, 0.015]	−**1.59 [**−**3.15,** −**0.023]**
	UBCR	−0.0020 [−0.0048, 0.00087]	−0.0025 [−0.0054, 0.00034]	−0.0021 [−0.0049, 0.00073]	−0.0018 [−0.0046, 0.0011]
	Log (UBCR + 1)	−**0.23 [**−**0.43,** −**0.028]**	−**0.29 [**−**0.50,** −**0.083]**	−**0.23 [**−**0.43,** −**0.027]**	−0.21 [−0.41, 0.0010]
Fructose	Scaled (mg/mL)	−**0.35 [**−**0.62,** −**0.078]**	−**0.34 [**−**0.61,** −**0.071]**	−0.18 [−0.45, 0.085]	−0.17 [−0.44, 0.10]
	UBCR	−**0.00035 [**−**0.00065,** −**0.000057]**	−**0.00037 [**−**0.00067,** −**0.000074]**	−0.00018 [−0.00048, 0.00012]	−0.00015 [−0.00045, 0.00015]
	Log (UBCR + 1)	−**0.13 [**−**0.22,** −**0.042]**	−**0.15 [**−**0.24,** −**0.061]**	−**0.12 [**−**0.21,** −**0.030]**	−**0.11 [**−**0.21,** −**0.022]**
Sucrose	Scaled (mg/mL)	−2.07 [−5.03, 0.90]	−1.98 [−4.94, 0.98]	−2.91 [−5.84, 0.03]	−2.91 [−5.87, 0.05]
	UBCR	−0.0027 [−0.0079, 0.0024]	−0.0031 [−0.0082, 0.0021]	−0.0039 [−0.0090, 0.0012]	−0.0038 [−0.0089, 0.0014]
	Log (UBCR + 1)	−0.073 [−0.18, 0.033]	−0.082 [−0.19, 0.025]	−**0.11 [**−**0.21,** −**0.0023]**	−0.10 [−0.21, 0.0059]

Abbreviations: CI, confidence interval; LNCS, low-and no-calorie sweeteners; UBCR, urinary biomarker-to-creatinine ratio; WHtR, waist-to-height ratio.

1 All adjusted models were adjusted for age, sex, fruit consumption frequency, vegetable consumption frequency, and screen time hours.

2 Total LNCS = acesulfame + saccharin + sucralose + cyclamate + steviol glucuronide.

3 LNCS and sugar concentration units were converted from μg/mL to mg/mL to present regression coefficients in interpretable ranges.

4 UBCR is a unitless ratio.

5 Log (UBCR + 1) was applied to normalize the distribution and address urine dilution; the addition of 1 accounts for 0 values.

6 Total sugar = glucose + fructose + sucrose.

Regarding urinary sugars, inverse associations with BMI z-score were observed for total sugars and fructose, which remained significant in multivariable models [total sugars β_mg/mL_: −0.32 (95% CI: −0.57, −0.08); fructose β_mg/mL_: −0.34 (95% CI: −0.61, −0.071)]. Similar results were observed when urinary sugars were expressed as UBCR. Glucose was inversely associated with BMI z-score in multivariable models, but only when log-transformed UBCR was used as exposure [β_Log-UBCR_: −0.29 (95% CI: −0.50, −0.083)].

For WHtR z-score, total sugars showed inverse associations in both crude and multivariable models when log-transformed UBCR was applied. Glucose (mg/mL) and fructose (log-transformed UBCR) were also inversely associated with WHtR z-score in adjusted models. Sucrose was inversely associated with WHtR z-score in the unadjusted model, but this association was slightly attenuated after adjusting for confounders [β_Log-UBCR_: −0.10 (95% CI: −0.21, 0.0059)].

**FIGURE 1:**
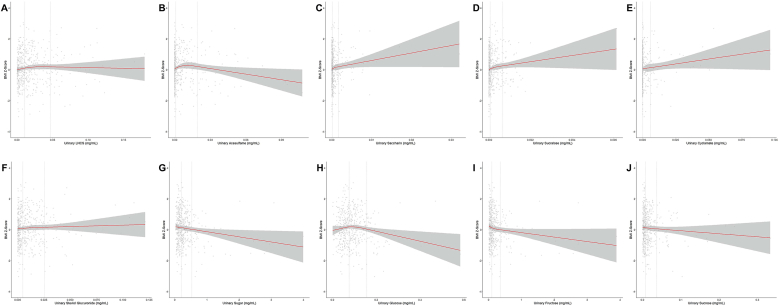
Restricted cubic spline models (3-knot) for associations between urinary low-and no-calorie sweetener (LNCS) and sugar concentrations (mg/mL) and BMI z-score. All models were adjusted for age, sex, fruit consumption frequency, vegetable consumption frequency, and screen time hours. Dashed lines indicate the locations of the 2nd and 3rd knots, corresponding to the 50th and 90th percentiles of the exposure variable by default. (**A**) Total LNCS, *P* for nonlinearity = 0.24; (**B**) acesulfame, *P* for nonlinearity = **0.02**; (**C**) saccharin, *P* for nonlinearity = 0.65; (**D**) sucralose, *P* for nonlinearity = 0.58; (**E**) cyclamate, *P* for nonlinearity = 0.94; (**F**) steviol glucuronide, *P* for nonlinearity = 0.89; (**G**) total sugar, *P* for nonlinearity = 0.96; (**H**) glucose, *P* for nonlinearity = **0.02**; (**I**) fructose, *P* for nonlinearity = 0.43; (**J**) sucrose, *P* for nonlinearity = 0.95.

**FIGURE 2:**
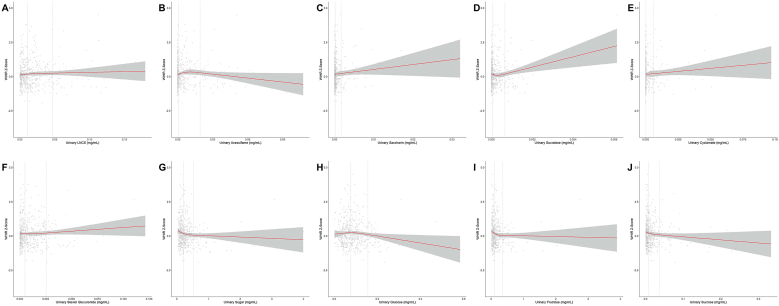
Restricted cubic spline models (3-knot) for associations between urinary low-and no-calorie sweetener (LNCS) and sugar concentrations (mg/mL) and waist-to-height ratio (WHtR) z-score. All models were adjusted for age, sex, fruit consumption frequency, vegetable consumption frequency, and screen time hours. Dashed lines indicate the locations of the 2nd and 3rd knots, corresponding to the 50th and 90th percentiles of the exposure variable by default. (**A**) Total LNCS, *P* for nonlinearity = 0.58; (**B**) acesulfame, *P* for nonlinearity = **0.03**; (**C**) saccharin, *P* for nonlinearity = 0.69; (**D**) sucralose, *P* for nonlinearity = **0.048**; (**E**) cyclamate, *P* for nonlinearity = 0.88; (**F**) steviol glucuronide, *P* for nonlinearity = 0.98; (**G**) total sugar, *P* for nonlinearity = 0.09; (**H**) glucose, *P* for nonlinearity = 0.26; (**I**) fructose, *P* for nonlinearity = 0.051; (**J**) sucrose, *P* for nonlinearity = 0.32.

Multivariable RCS analyses indicated no significant evidence of nonlinearity for most associations between urinary LNCS and sugar concentrations with BMI z-score (Figure 1A–J) and WHtR z-score (Figure 2A–J). However, an inverted J-shaped association was observed between glucose and BMI z-score (Figure 1H; *P* for nonlinearity = 0.02). Similar nonlinear relationships were observed for acesulfame with BMI z-score (Figure 1B; *P* for nonlinearity = 0.02) and WHtR z-score (Figure 2B; *P* for nonlinearity = 0.03), with downward trends shown at higher urinary acesulfame concentrations. In contrast, an upward trend was observed for sucralose with WHtR z-score as its concentration increased (Figure 2D; *P* for nonlinearity = 0.048).

As additional analyses, associations of total LNCS and total sugar concentrations with BMI and WHtR z-scores were assessed separately within age and sex subgroups. However, no significant interactions were observed (Supplemental Table 2).

## Discussion

We investigated the associations between urinary LNCS and sugars with BMI and WHtR z-scores among a population cohort sample of 500 Dutch children and adolescents, introducing biomarkers to assess dietary exposure. Taking potential confounding variables into account, urinary saccharin was positively associated with BMI z-score, whereas urinary sucralose showed positive associations with both BMI and WHtR z-scores. In contrast, total urinary sugars, glucose, and fructose were inversely associated with BMI and WHtR z-scores. Our study further revealed an inverted J-shaped relationship between urinary glucose concentration and BMI z-score. Similar nonlinear trends were observed for acesulfame with BMI and WHtR z-scores.

We observed positive cross-sectional associations for urinary saccharin with general obesity and for urinary sucralose with indicators of both general and central obesity. This suggests either differences in how obesity indicators respond to LNCS exposures, and/or points to variation in eating behaviors across obesity phenotypes. Previous observational studies have reported that LNCSB consumption was associated with a higher prevalence of general obesity in children and adolescents [15,16,20], as further confirmed by a meta-analysis [11]. Particularly, a large-scale cohort study among 3227 children and adolescents aged 9–17 y found that each 100 mL increase in LNCSB consumption was associated with increased odds of both general obesity [odds ratio 1.14 (95% CI: 1.06, 1.23)] and central obesity [odds ratio 1.12 (95% CI: 1.03, 1.23)] [20]. However, other observational studies have reported no association [13,18,23]. In comparison, a recent meta-analysis of 4 randomized controlled trials involving children and adolescents aged 4–18 y concluded that replacing SSBs with beverages containing a mixture of LNCS or sucralose resulted in less BMI gain (not weight loss) compared with sustained SSB intake [mean difference: −0.114 (95% CI: −0.207, −0.021)] [49].

Independent of beverage sources, however, most epidemiological studies may not account for LNCS from other products, such as saccharin in ready-to-eat meals, sauces, and condiments, or sucralose in chewing gum, candies, jams, and canned foods [50]. Our findings of positive associations between urinary saccharin and sucralose and BMI and WHtR z-scores agree with observational studies on LNCSBs and extend them to LNCS exposure from nonbeverage sources. Given this, experimental research on individual LNCS may provide further insights into our findings. Saccharin intake has been linked to impaired glucose homeostasis and obesity-inducing effects in rodent models [51,52]. Similarly, sucralose exposure has been shown to promote adipogenesis in human subcutaneous adipose tissue-derived mesenchymal stromal cells [53]. Despite these metabolic implications, the possibility of reverse causality is equally plausible in our study, as children with overweight or obesity are often advised to limit sugar intake, possibly replacing it with LNCS–containing foods and beverages [12]. Longitudinal studies on changes in urinary LNCS are needed to further elucidate this issue.

Total urinary sugars, glucose, and fructose were inversely associated with general and central obesity in our study. This may seem counterintuitive compared with previous observational studies [14,15,21] as well as systematic reviews and meta-analyses [7,43,54], in which positive associations were reported between SSB consumption and obesity risk among children and adolescents. However, a cross-sectional survey of 3860 Dutch children and adolescents (aged 10–16 y) indicated that intake of sweets was significantly associated with a lower prevalence of overweight and obesity [odds ratio 0.87 (95% CI: 0.81, 0.94)] [55]. Similar results were observed in EU Childhood Obesity Project, where an inverse association was reported between sugar intake and BMI z-score [odds ratio 0.743 (95% CI: 0.611, 0.903)] among 809 children (aged 2–8 y) [56]. In addition, a meta-analysis of 11 cross-sectional studies involving 177,260 children and adolescents (aged 2–18 y) found that each additional weekly serving of confectionery was associated with a 13% decrease in the odds of overweight or obesity [57]. Although the underreporting of sugary foods among obese children has been frequently discussed [58], our use of objective biomarkers suggests that social desirability bias alone does not fully explain the inverse associations and may instead reflect actual dietary differences.

Interestingly, we observed an inverted J-shaped relationship between urinary glucose and BMI z-score, where children with higher BMI z-scores excreted less glucose, whereas those with lower BMI z-scores excreted more. At higher urinary glucose concentration, the downward trend may reflect children and adolescents with normal weight without metabolic disturbances [59]. Physiologically, glucose is excreted in urine when blood glucose concentrations exceed the renal threshold (around 180 mg/dL), which is a temporary condition in healthy individuals after excessive sugar consumption, that is, alimentary glycosuria [60]. At lower urinary glucose concentration, the nonlinear trend may reflect a mixture of situations. For example, children and adolescents with higher BMI z-scores or those seeking a slimmer body image may intentionally reduce sugar intake, especially glucose and sucrose from added sugars, by shifting toward LNCS–containing foods [12,61]. It is also possible that a small number of children and adolescents were in the early stages of insulin resistance, which is increasingly common in children and adolescents with obesity [62]. In that case, increased expression of sodium–glucose cotransporter 2 enhances renal glucose reabsorption from glomerular filtrate, contributing to reduced urinary glucose excretion [60,63]. However, a similar trend was not reported previously, suggesting the necessity of further studies combining cardiometabolic markers and urinary biomarkers to confirm the potential link between insulin resistance and urinary glucose excretion in children and adolescents.

We also identified nonlinear associations between acesulfame and both BMI and WHtR z-scores, with downward trends observed at higher urinary acesulfame concentrations. Experimental studies have linked acesulfame to adverse effects, including disturbances in gut microbiota and dysregulation of lipid metabolism [64,65]. A possible explanation for our findings is that acesulfame may be derived from food sources distinct from those of other LNCS. Although data specifying LNCS–containing foods in Dutch dietary databases are limited, research from Spain assessing LNCS in food products indicated that acesulfame was present more often in sports drinks, vegetable drinks, and breakfast cereals compared with saccharin and sucralose [50]. It is likely that these products have higher fiber content and are preferred by physically active individuals with lower BMI and healthier dietary habits (for example, eating breakfast) [66,67]. However, our study was unable to distinguish between food sources. Because acesulfame is often paired with other LNCS in food, it remains to be explored whether the observed association reflects the independent effect of acesulfame or joint effects of coingested sweeteners.

In this study, spot urine biomarkers were used to assess LNCS and sugar exposures among children and adolescents, adjusting for urinary dilution with UBCR. Although 24-h urine collection is considered the gold standard for estimating dietary intake, its feasibility in pediatric populations and large-scale epidemiological research is limited due to economic and participant burden [68]. In contrast, spot urine collection offers a more practical option, yet it is influenced by diurnal fluctuations, hydration status, and urinary volume, and reflects only a partial snapshot of recent eating episodes [68,69]. To better approximate daily dietary exposure, previous studies, mostly on sodium and potassium, have suggested predicting 24-h excretion from spot urine biomarkers and creatinine using Tanaka’s equation [70,71]. However, the predicted values from spot urine may not fully align with the actual 24-h biomarker and creatinine excretion because of limited information on biomarker excretion kinetics and variations in creatinine excretion across ethnicities, age, and sex groups [69]. Alternatively, UBCR was applied to minimize influences because of variability in hydration status and single urine sample collection [72]. The I.Family study has reported a moderate validity coefficient (0.411) for log-transformed UBCR-adjusted sucrose and fructose from spot urine biomarkers with total sugar intake from a 24-h dietary recall among European children and adolescents (aged 5–18 y) [73]. Nevertheless, creatinine correction may introduce new uncertainty in the studied associations because of its potential association with obesity [74]. It may be of future interest to explore the reliability of using spot urine LNCS and sugars to predict 24-h excretion as well as validate the application of UBCR in examining associations between urinary biomarker levels and health outcomes.

This study is one of the first to apply urinary biomarkers in examining the associations of LNCS and sugar with obesity outcomes in children and adolescents. The use of urinary biomarkers provides an objective and direct measure to quantify LNCS and sugars individually. The study also benefits from a relatively large sample size of 500 participants, which is substantial compared with similar research utilizing nutritional biomarkers [75].

Several limitations should be considered when interpreting the findings of this study. First, although UBCR was used for dilution adjustment, the method relies on the assumption of stable creatine excretion and may therefore be less precise than alternatives such as urine flow rate and osmolality [69]. Second, despite a biomarker approach offering a certain level of objectivity for assessing exposure to sweeteners, it is more complicated for sugars [76]. For instance, urinary glucose excretion depends on reabsorption in the kidney and is not specific to dietary intake, as it may also be derived from noncarbohydrate substrates via gluconeogenesis or from the breakdown of other sugars. Third, our findings may not accurately reflect the habitual consumption patterns of LNCS and sugars, as repeated or 24-h urine samples were not available. The lack of dietary intake data also limited our ability to compare methods (that is, self-reported intake compared with urinary excretion), and investigate food matrices and sources, especially natural and added sugars. Moreover, although we controlled for several important covariates, we were not able to adjust for residual confounding from other relevant factors, including socioeconomic status, family history, or physical activity level. Furthermore, the results may not be generalizable to other ethnicities and countries with distinct consumption behaviors and regulations regarding sugar and sweetener use in food products. Finally, as indicated earlier, causal inference cannot be obtained because of cross-sectional design. However, the use of RCS enabled visualization of trends, providing valuable insights into the nature of these associations.

In conclusion, in a cohort of children and adolescents from the general population, we observed positive associations for urinary saccharin with BMI z-score and sucralose with both BMI and WHtR z-scores. Inverse associations were found for total urinary sugar, glucose, and fructose with BMI and WHtR z-scores, particularly when assessed relative to urinary creatinine. These findings are likely to reflect differences in food and beverage consumption patterns among children and adolescents and emphasize the value of biomarkers for more objective dietary assessment.

## Author contributions

The authors’ responsibilities were as follows – XC, EB-B, MB, EJMF: developed the research questions and planned the analysis; NDN: conducted random sampling; GdG performed the laboratory analysis; XC: analyzed the data and drafted the manuscript; EB-B, MB, EJMF: provided significant consultation; and all authors: contributed to data interpretation and critical revision of the manuscript.

## Data availability

Data are available from the Lifelines Cohort study upon reasonable request. For access to the data that support the findings of this study, the Lifelines research office can be contacted via www.lifelines.nl/researcher.

## Funding

This European Union project under the acronym “SWEET” has received funding from the European Union’s Horizon 2020 research and innovation program under grant agreement No 774293. The Lifelines Cohort Study initiative has been made possible by a subsidy from the Dutch Ministry of Health, Welfare and Sport, the Dutch Ministry of Economic Affairs, the University Medical Center Groningen (UMCG), Groningen University, and the Provinces in the North of the Netherlands (Drenthe, Friesland, Groningen). The funders had no role in the study design, data collection, data analysis and interpretation, writing the report, or the decision to submit the article for publication.

## Conflicts of interest

The University of Leeds received income from consultees of JCGH with Allurion and Dupont IFF. JCGH and JAH receive research funding from the American Beverage Association. AR holds shares and is employed at Novo Nordisk A/S, Søborg, Denmark and has received honoraria from International Sweeteners Association, Nestlé, and Unilever. All other authors report no conflicts of interest.
