# Supplementary material for: Urinary Sweeteners and Sugars in Relation to Childhood Obesity: The SWEET Project
Source: J Nutr. 2025 Oct 10;155(12):4476–85. doi: 10.1016/j.tjnut.2025.10.019 (PMC12799442; doi:10.1016/j.tjnut.2025.10.019)
Supplement: multimedia component 1 [file mmc1.docx]

**Supplementary Table 1** BMI z-score and WHtR z-score stratified by age and sex groups.

| **Outcomes** | **All**^1^ |  | **Age group**^2^ |  |  | **Sex group** |  |
| --- | --- | --- | --- | --- | --- | --- | --- |
|  |  | **Children** | **Adolescents** | **P-value**^3^ | **Girls** | **Boys** | **P-value**^3^ |
| BMI z-score | 0.10 (1.0) | 0.17 (1.1) | 0.04 (1.0) | 0.18 | 0.22 (0.9) | -0.004 (1.1) | **0.02** |
| Overweight (%)^4^ | 93 (18.6) | 50 (20.0) | 43 (17.2) | - | 56 (22.5) | 37 (14.7) | - |
| Obesity (%) | 14 (2.8) | 10 (4.0) | 4 (1.6) | - | 4 (1.6) | 10 (4.0) | - |
| WHtR z-score | 0.16 (1.0) | 0.12 (1.1) | 0.20 (1.0) | 0.40 | 0.21 (0.9) | 0.10 (1.1) | 0.26 |
| Central obesity (%)^5^ | 27 (5.4) | 16 (6.4) | 11 (4.4) |  | 13 (5.2) | 14 (5.6) |  |

Abbreviations: BMI, body mass index; WHtR, waist to height ratio; SD, standard deviation.

^1^ Data are mean (sd) or n (%).

^2^ Age group: children (aged 8-14, n=250) and adolescents (aged 15-17, n=250).

^3^ P-value derived from t-test.

^4^ Overweight was defined as 1 ≤ BMI z-score < 2, and obesity as BMI z-score ≥ 2.

^5^ Central obesity was defined as WHtR ≥ 0.5.

**Supplementary Table 2** Multivariable associations for urinary LNCS and sugar concentrations (mg/mL) with BMI z-score and WHtR z-score, stratified by age and sex.

|  | **Total LNCS**^1,2^ | **Total Sugar**^1,3^ |
| --- | --- | --- |
| **BMI z-score** |  |  |
| *Age group* |  |  |
| Children | 1.55 [-4.05, 7.16] | **-0.37 [-0.71, -0.03]** |
| Adolescents | 1.62 [-3.63, 6.87] | -0.25 [-0.61, 0.10] |
| P for interaction | 0.88 | 0.66 |
| *Sex group* |  |  |
| Boys | 0.46 [-5.43, 6.35] | **-0.37 [-0.68, -0.06]** |
| Girls | 3.07 [-1.85, 8.00] | -0.20 [-0.62, 0.21] |
| P for interaction | 0.48 | 0.60 |
| **WHtR z-score** |  |  |
| *Age group* |  |  |
| Children | -0.07 [-0.32, 0.17] | -0.008 [-0.023, 0.007] |
| Adolescents | 0.15 [-0.07, 0.37] | -0.006 [-0.022, 0.009] |
| P for interaction | 0.17 | 0.82 |
| *Sex group* |  |  |
| Boys | -0.006 [-0.26, 0.25] | -0.008 [-0.021, 0.006] |
| Girls | 0.07 [-0.14, 0.28] | -0.006 [-0.024, 0.012] |
| P for interaction | 0.60 | 0.95 |

Abbreviations: BMI, body mass index; WHtR, waist to height ratio; CI, confidence interval.

^1^ All models were adjusted for age or sex (depending on the subgroup), fruit consumption frequency, vegetable consumption frequency, and screen time hours. Regression coefficients (β) and 95% CIs were reported.

^2^ Total urinary LNCS = acesulfame + saccharin + sucralose + cyclamate + steviol glucuronide.

^3^ Total sugar = glucose + fructose + sucrose.
